# Cancer—A Few Suggestions

**Published:** 1905-12

**Authors:** R. R. Kime

**Affiliations:** Atlanta, Ga.


### CANCER—A FEW SUGGESTIONS.

By R. R. KIME, M.D.,

ATLANTA, GA.

Cancer is ever with us and ever securing new victims in its onward march from year to year.

The researches of the past have failed to demonstrate its exact pathology or a specific treatment. Most of the accumulated evidence tends to demonstrate that it is due to a germ or cellular infiltration.

In the treatment of cancer surgery offers early and complete removal as its best contribution. The X-ray and radium have not fulfilled their expectations. It will require time and experience to demonstrate their limitations. Galvanic electricity or electrolysis is in its experimental stage. Escharotics have their field of usefulness, but all fall short of universal applicability.

It is not probable that any one remedy or procedure will be universally applicable because of the different locations, different organs and tissues that are involved by different forms of malignant disease. Until we have a positive demonstration of the cause of cancer our searches for a remedy or relief must be experimental or empyrical.

If it be true that cancer is due to a parasite, protozoa or cellular infiltration any remedy that will destroy or prevent the development of either without the destruction of living healthy tissue may prove of benefit. As a contribution along this line I offer the following as a palliative measure and a possible relief in some cases until something more efficient is found:

A combination of salicylic acid, carbolic acid and gum camphor, adding at certain stages in the treatment of some cases an equal volume of balsam of Peru.

This combination is strongly antiseptic, decidedly germicidal and a destroyer of low forms of cell development. It does not corrode nor destroy healthy tissue if properly made and properly used. It has the power of penetration of tissue and is taken up by the lymphatics. A point that should not be forgotten in these cases is to secure free drainage so as to eliminate rather than absorb toxic material.

Where there is an excessive cancer growth, fungoid in character, making pressure, preventing drainage, it should be removed so as to give free drainage, lesson amount of cancer material and provide for absorption of remedy used provided it is not possible to remove all the cancerous growth. Especially is this true of uterine cancer. In such cases high amputation of cervix, curetting out interior of uterus, applying the actual cautery, following with the local treatment is at least palliative and in some possibly curative in results.

The details of treatment have not been worked out sufficiently to justify their presentation at this time, neither are its claims sufficiently established. I desire more cases to test its merits. Epithelioma, uterine cancer and those with ulcerating surfaces are preferred, especially inoperable cases and uterine cancer before hysterectomy is performed. Physicians having such cases they do not care to treat, I would appreciate an opportunity of trying this method to see what can be done for their relief. Any cases referred will be duly appreciated.

509-11 English-American Building.

